# Supplementary material for: Discovering genetic interactions bridging pathways in genome-wide association studies
Source: Nat Commun. 2019 Sep 19;10:4274. doi: 10.1038/s41467-019-12131-7 (PMC6753138; doi:10.1038/s41467-019-12131-7)
Supplement: Supplementary file 1 — Supplementary Information [file 41467_2019_12131_MOESM1_ESM.pdf]

Supplementary Information

Discovering genetic interactions bridging pathways in genome-wide association studies

Fang and Wang et.al.

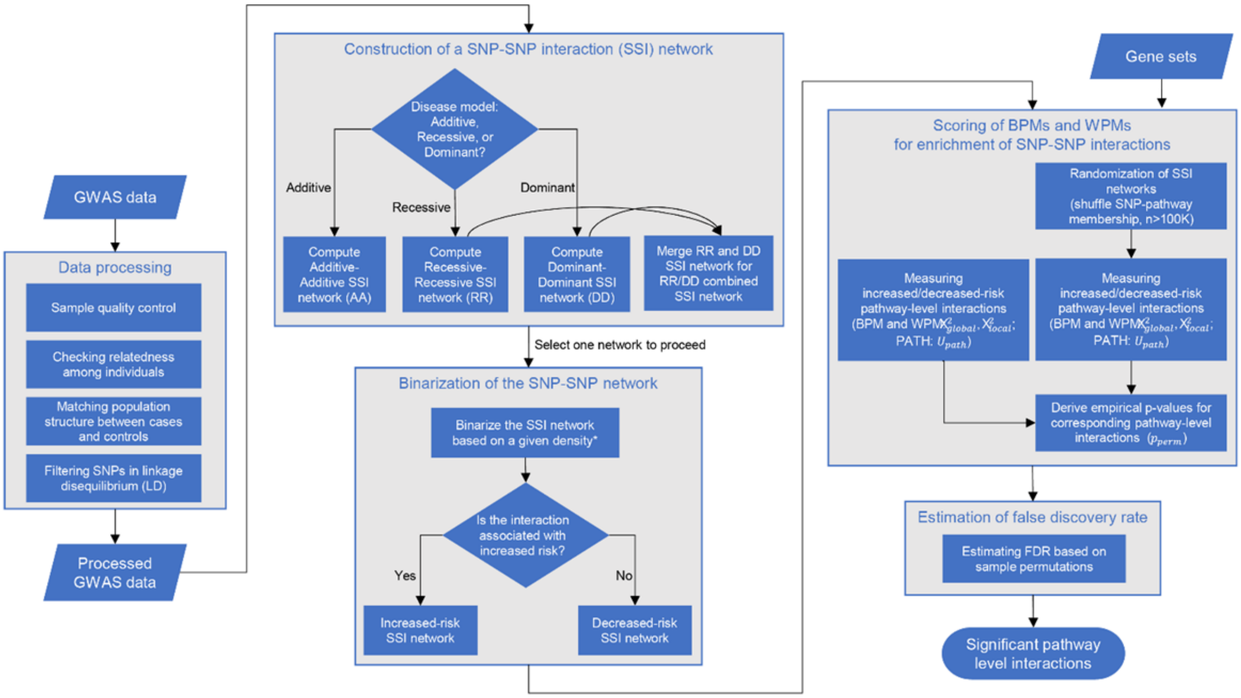

* Binarization density is determined based on a pilot run (See method)

Supplementary Figure 1: Detailed view of the BridGE method for detecting genetic interactions from GWAS data.

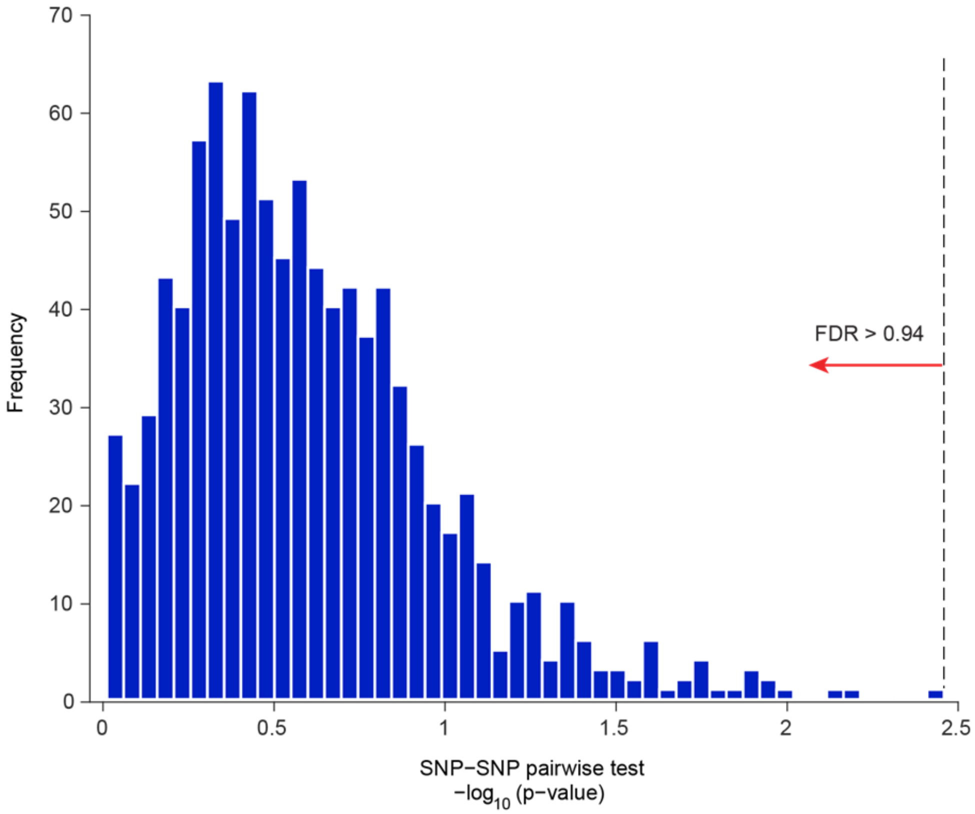

Supplementary Figure 2: Distribution of p-values from individual tests for pairwise SNP-SNP interactions for discovered Parkinson's disease BPM. SNP pairs supporting the pathway-pathway interaction between the Golgi associated vesicle biogenesis gene set (Reactome) and Fc epsilon receptor I signaling pathway (KEGG) discovered from the PD-NIA Parkinson's disease cohort were evaluated for association with PD based on a recessive and dominant disease model. The distribution of maximum $-\log_{10}$ hypergeometric test p-value of the two models for each SNP pair is plotted. None of the SNP pairs are significant after multiple hypothesis correction (dashed line at the most significant SNP-SNP pair corresponds to FDR=0.94).

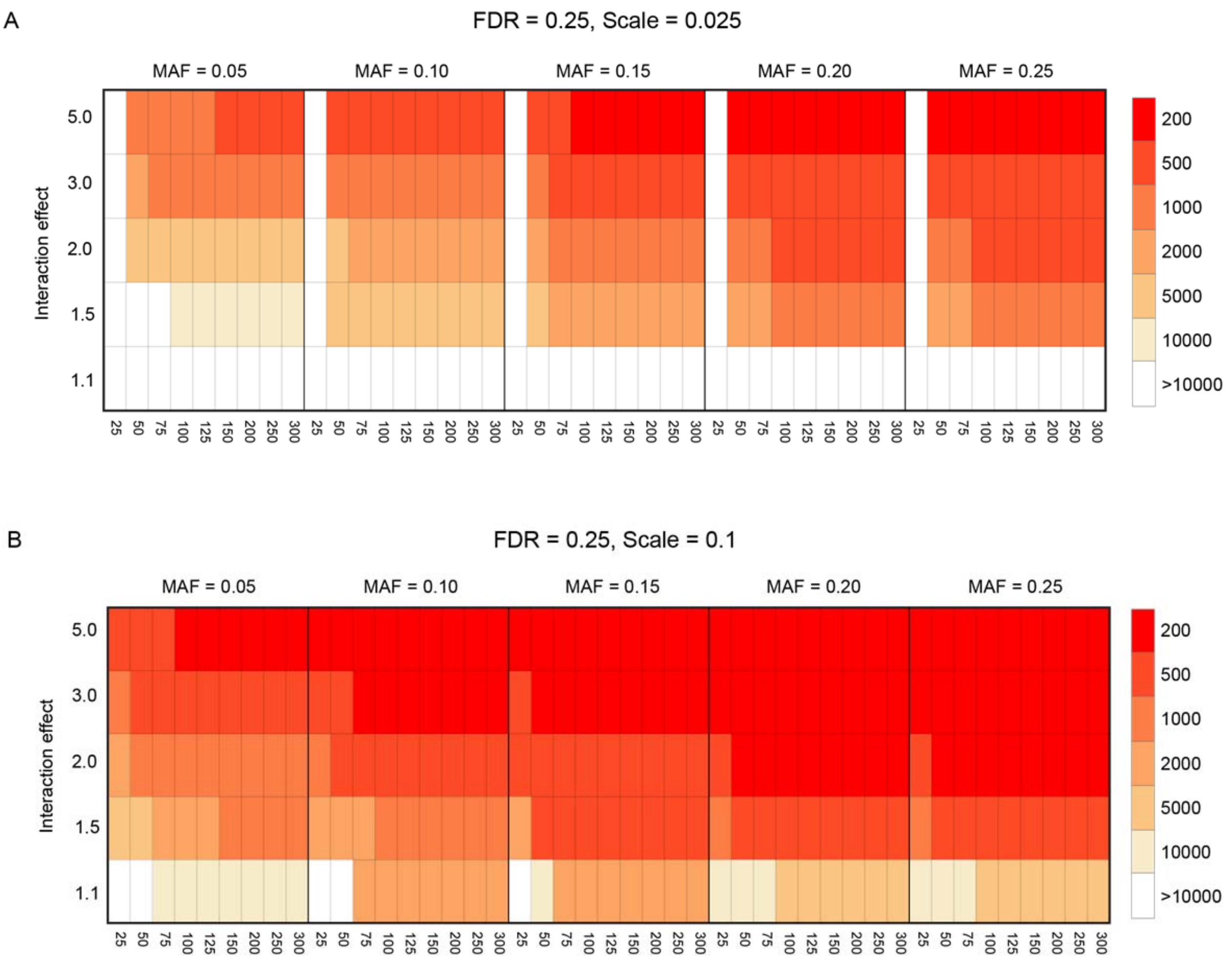

Supplementary Figure 3: Power analysis of the effect of minor allele frequency (MAF), BPM size, interaction effect size, and sample size on the discovery of between-pathway interactions. The plot is same as Fig. 6, but the biological densities used are 2.5% (a) and 10% (b).

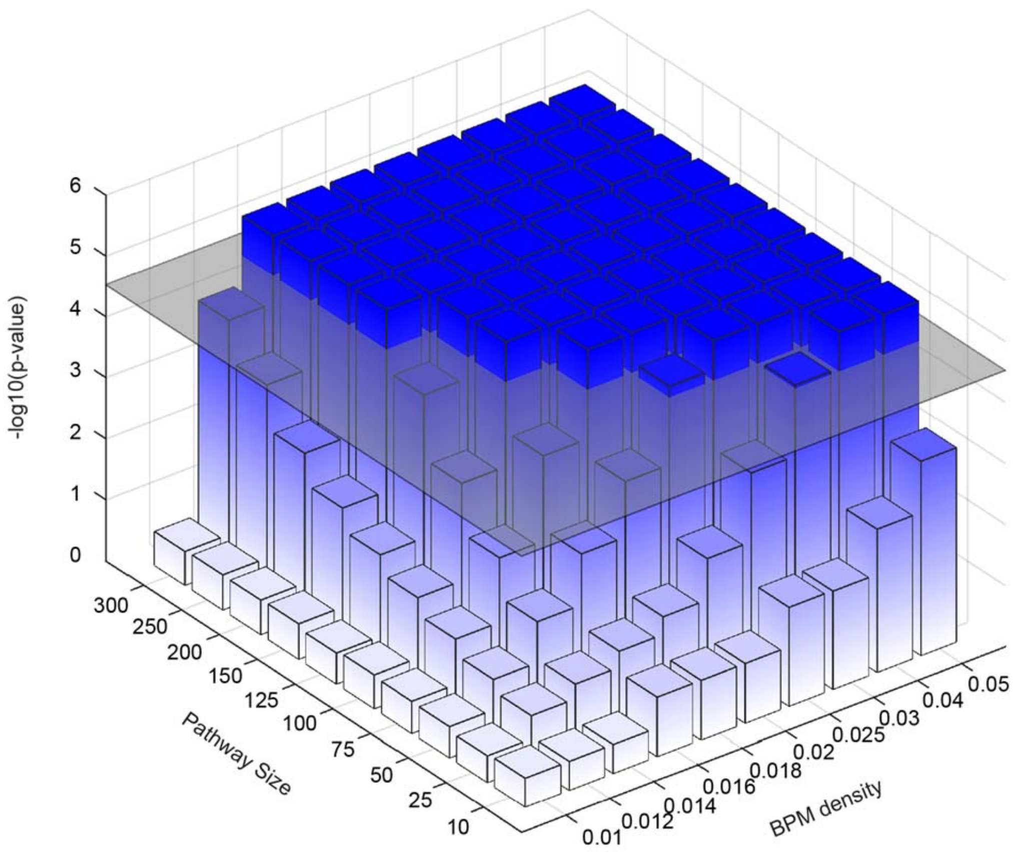

Supplementary Figure 4: Power simulation of the effect of pathway size and interaction density on the discovery of between-pathway interactions. The BPM significance ($-log_{10}$ p-value derived from 150,000 SNP permutations) is plotted for 100 embedded BPMs of different sizes and SNP-SNP interaction densities (online method). The gray plane indicates the p-value cutoff corresponding to the average SNP permutation p-values (p = 3.0×10^{-5}) of the significant BPM discoveries across all GWAS cohorts (FDR≤0.25). Bars exceeding this plane represent BPMs that would have been discovered in this cohort and provide an estimate of sensitivity of the approach.

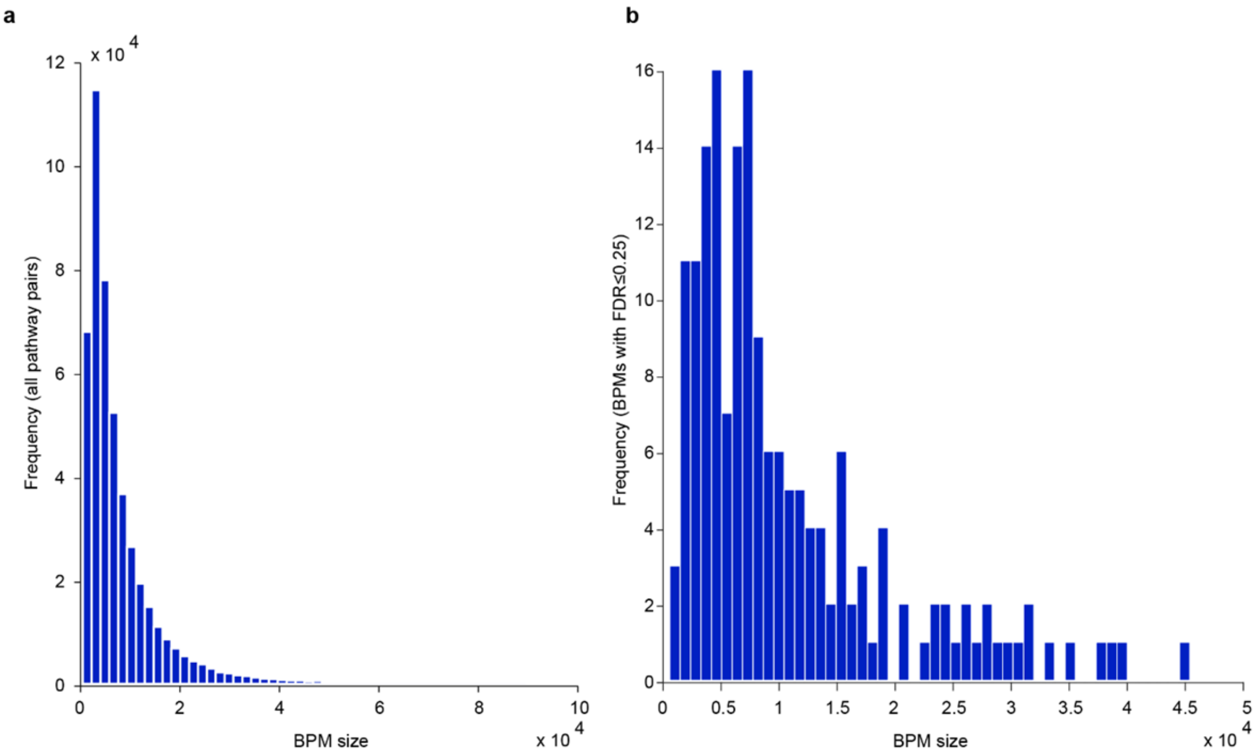

Supplementary Figure 5: Distribution of sizes for discovered BPMs. The size of each candidate BPM was measured as the total number of possible SNP-SNP pairs between the two pathways. The distribution of sizes of all possible pathway-pathway pairs is plotted in (a) and only significant BPMs (FDR ≤ 0.25) from the PD-NIA cohort are plotted in (b). BPMs discovered by BridGE span a large range of sizes.

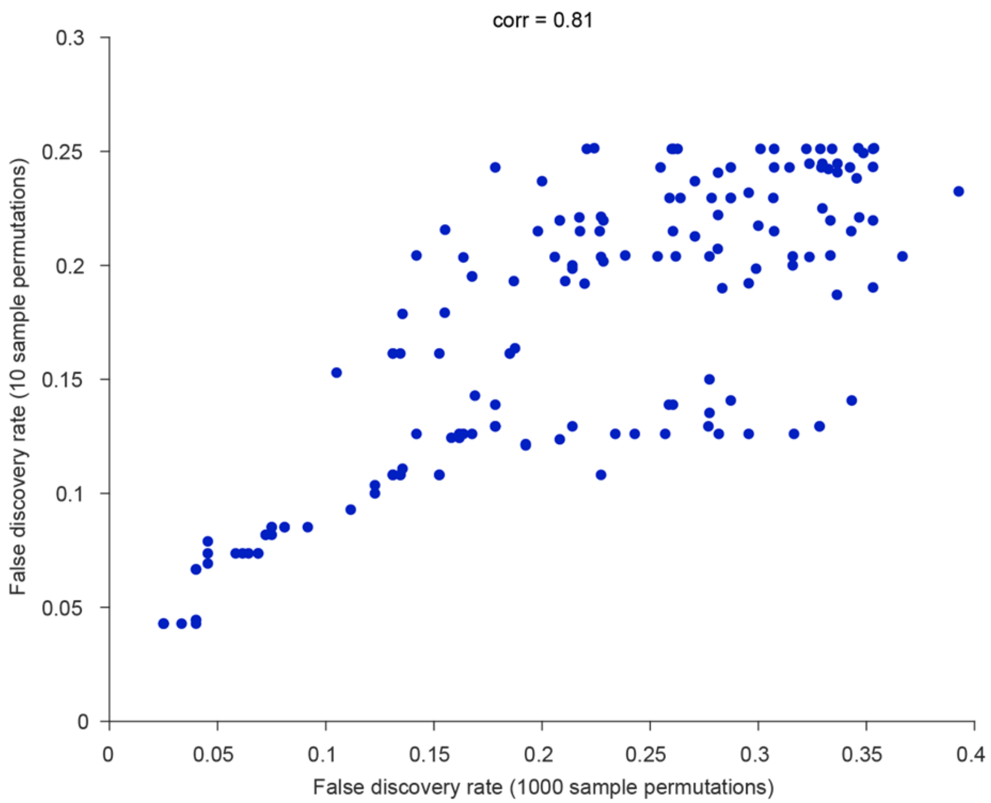

Supplementary Figure 6: Comparison of false discovery rates derived from 10 sample permutations vs. 1000 sample permutations using PD-NIA dataset. BPMs that are significant (FDR≤0.25) based on either 10 sample permutations or 1000 permutations were plotted to show the agreement between two permutations.

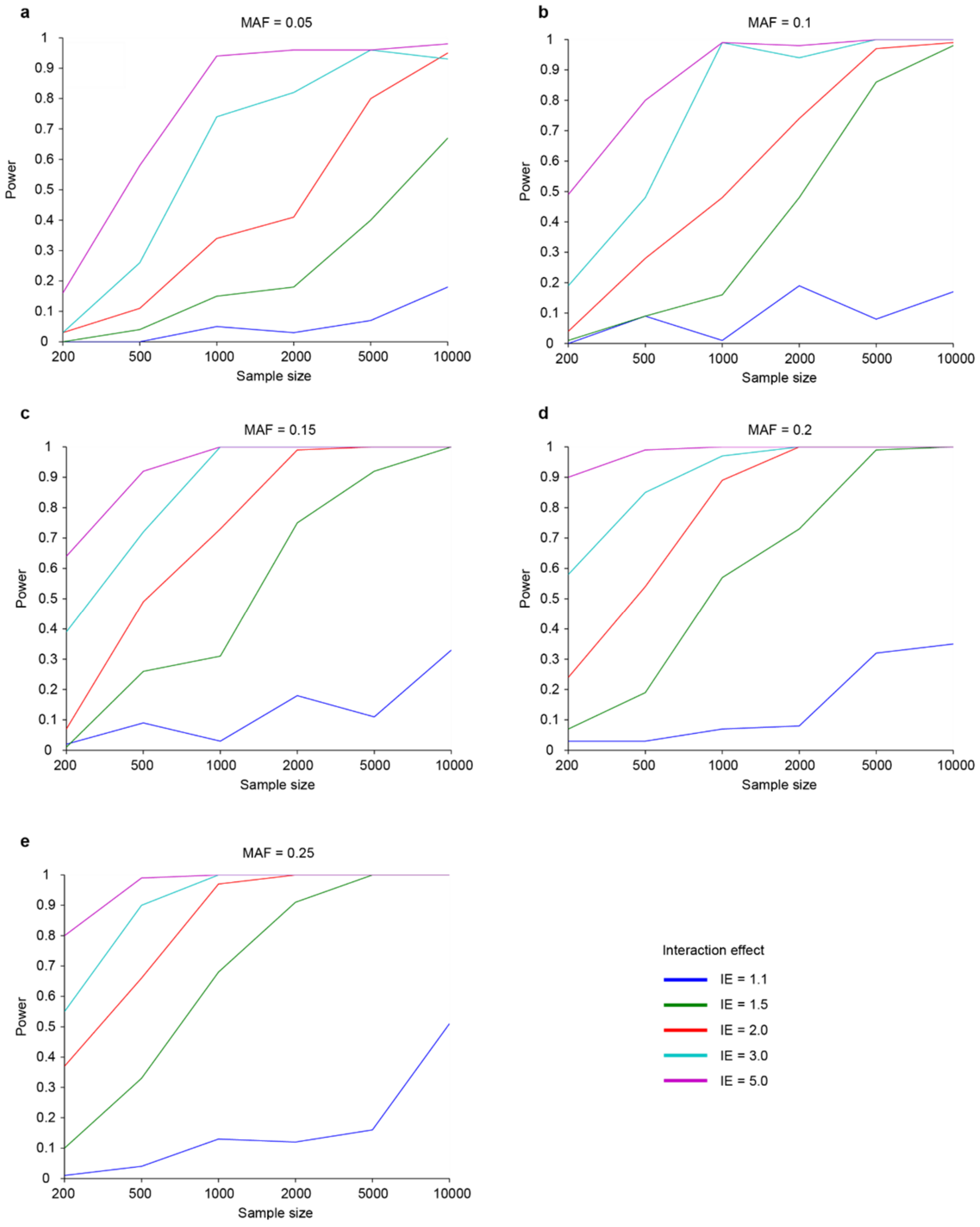

Supplementary Figure 7: Power simulation of the effect of sample size, interaction effect size (IE) and minor allele frequency on the discovery of SNP-SNP interactions0, The discovery rates of 100 embedded SNP-SNP interactions in the synthetic datasets with different sample sizes were plotted and colored with corresponding interaction effect size. Each subplot is corresponded to a different minor allele frequency assumption: (a) MAF=0.05, (b) MAF=0.1, (c) MAF=0.15, (d) MAF=0.2, (e) MAF=0.25.

Supplementary Discussion

BridGE results on hypertension and type2 diabetes

Although we did not conduct replication analyses for hypertension or type 2 diabetes, we found that many of the pathways involved in interactions from the discovery cohorts were also highly relevant to the corresponding disease. For example, in the hypertension cohort, we identified a risk-associated BPM interaction involving hypoxia inducible factor (HIF) signaling, whose aberrant expression has been previously associated with hypertension[1]. Two BPMs and one WPM, all associated with increased risk, involved the Rho cell motility signaling pathway, which has been previously implicated in the pathogenesis of hypertension[2]. For type 2 diabetes, we discovered BPMs associated with protective effects involving an autoimmune thyroid disease gene set, glycosaminoglycan biosynthesis, and the mTOR signaling pathway, all of which have strong links to diabetes[3,4,5].

Additional Acknowledgements

The genome-wide association datasets (PD-NIA, PD-NGRC, SZ-GAIN, BC-CGEMS-EUR, BC-MCS-JPN, BC-MCS-LTN, HT-eMERGE, ProC-CGEMS, ProC-BPC3 and PanC-PanScan) used in this study were obtained from https://www.ncbi.nlm.nih.gov/gap through dbGaP accession numbers: phs000089.v3.p2, phs000196.v3.p1, phs000021.v3.p2, phs000147.v3.p1, phs000517.v3.p1, phs000297.v1.p1, phs000207.v1.p1, phs000812.v1.p1, and phs000206.v5.p3. We acknowledge the Contributing Investigators who submitted data from their original study to dbGaP, the primary funding organization that supported the Contributing Investigators, and the NIH data repository.

The genome-wide association datasets (SZ-GAIN, HT-WTCCC, T2D-WTCCC) used in this study were provided by Wellcome Trust Case Control Consortium through Dataset Accession numbers: EGAD00000000006, EGAD00000000009 and EGAD00000000001 and EGAD00000000002. These were funded by the Wellcome Trust under award 076113 and a full list of the investigators who contributed to the generation of the data is available from www.wtccc.org.uk.

PD-NIA (phs000089.v3.p2):

The genotyping of samples was provided by the National Institute of Neurological Disorders and Stroke (NINDS). The dataset used for the analyses described in this manuscript were obtained from the NINDS Database found at https://www.ncbi.nlm.nih.gov/gap

PD-NGRC (phs000196.v3.p1):

This work utilized in part data from the NINDS DbGaP database from the CIDR:NGRC PARKINSON'S DISEASE STUDY.

SZ-GAIN (phs000021.v3.p2):

Funding support for the Genome-Wide Association of Schizophrenia Study was provided by the National Institute of Mental Health (R01 MH67257, R01 MH59588, R01 MH59571, R01 MH59565, R01 MH59587, R01 MH60870, R01 MH59566, R01 MH59586, R01 MH61675, R01 MH60879, R01 MH81800, U01 MH46276, U01 MH46289 U01 MH46318, U01 MH79469, and U01 MH79470) and the genotyping of samples was provided through the Genetic Association Information Network (GAIN). The datasets used for the analyses described in this manuscript were obtained from the database of Genotypes

and Phenotypes (dbGaP) found at http://www.ncbi.nlm.nih.gov/gap through dbGaP accession number phs000021.v3.p2. Samples and associated phenotype data for the Genome-Wide Association of Schizophrenia Study were provided by the Molecular Genetics of Schizophrenia Collaboration (PI: Pablo V. Gejman, Evanston Northwestern Healthcare (ENH) and Northwestern University, Evanston, IL, USA).

BC-CGEMS-EUR (phs000147.v3.p1):

This dataset was from the Cancer Genetic Markers of Susceptibility (CGEMS) Breast Cancer Genome-wide Association Study with dbGaP accession number phs000147.v3.p1.

BC-MCS-LTN, BC-MCS-JPN (phs000517.v3.p1):

The Multiethnic Cohort and the genotyping in this study were funded by grants from the National Institute of Health (CA63464, CA54281, CA098758, CA132839 and HG005922) and the Department of Defense Breast Cancer Research Program (W81XWH-08-1-0383).

HT-eMERGE (phs000297.v1.p1):

Group Health Cooperative/University of Washington – Funding support for Alzheimer's Disease Patient Registry (ADPR) and Adult Changes in Thought (ACT) study was provided by a U01 from the National Institute on Aging (Eric B. Larson, PI, U01AG006781). A gift from the 3M Corporation was used to expand the ACT cohort. DNA aliquots sufficient for GWAS from ADPR Probable AD cases, who had been enrolled in Genetic Differences in Alzheimer's Cases and Controls (Walter Kukull, PI, R01 AG007584) and obtained under that grant, were made available to eMERGE without charge. Funding support for genotyping, which was performed at Johns Hopkins University, was provided by the NIH (U01HG004438). Genome-wide association analyses were supported through a Cooperative Agreement from the National Human Genome Research Institute, U01HG004610 (Eric B. Larson, PI).

Mayo Clinic – Samples and associated genotype and phenotype data used in this study were provided by the Mayo Clinic. Funding support for the Mayo Clinic was provided through a cooperative agreement with the National Human Genome Research Institute (NHGRI), Grant #: UOIHG004599; and by grant HL75794 from the National Heart Lung and Blood Institute (NHLBI). Funding support for genotyping, which was performed at The Broad Institute, was provided by the NIH (U01HG004424).

Marshfield Clinic Research Foundation – Funding support for the Personalized Medicine Research Project (PMRP) was provided through a cooperative agreement (U01HG004608) with the National Human Genome Research Institute (NHGRI), with additional funding from the National Institute for General Medical Sciences (NIGMS) The samples used for PMRP analyses were obtained with funding from Marshfield Clinic, Health Resources Service Administration Office of Rural Health Policy grant number D1A RH00025, and Wisconsin Department of Commerce Technology Development Fund contract number TDF FYO10718. Funding support for genotyping, which was performed at Johns Hopkins University, was provided by the NIH (U01HG004438).

Northwestern University – Samples and data used in this study were provided by the NUgene Project (www.nugene.org). Funding support for the NUgene Project was provided by the Northwestern University's Center for Genetic Medicine, Northwestern University, and Northwestern Memorial Hospital. Assistance with phenotype harmonization was provided by the eMERGE Coordinating Center (Grant number U01HG04603). This study was funded through the NIH, NHGRI eMERGE Network (U01HG004609). Funding support for genotyping, which was performed at The Broad Institute, was provided by the NIH (U01HG004424).

Vanderbilt University - Funding support for the Vanderbilt Genome-Electronic Records (VGER) project was provided through a cooperative agreement (U01HG004603) with the National Human Genome Research Institute (NHGRI) with additional funding from the National Institute of General Medical Sciences (NIGMS). The dataset and samples used for the VGER analyses were obtained from Vanderbilt University Medical Center's BioVU, which is supported by institutional funding and by the Vanderbilt CTSA grant UL1RR024975 from NCRR/NIH. Funding support for genotyping, which was performed at The Broad Institute, was provided by the NIH (U01HG004424).

Assistance with phenotype harmonization and genotype data cleaning was provided by the eMERGE Administrative Coordinating Center (U01HG004603) and the National Center for Biotechnology Information (NCBI). The datasets used for the analyses described in this manuscript were obtained from dbGaP at http://www.ncbi.nlm.nih.gov/gap through dbGaP accession number phs000297.v1.p1.

ProC-CGEMS (phs000207.v1.p1):

This data was from the Cancer Genetic Markers of Susceptibility (CGEMS) Prostate Cancer Genome-Wide Association Study.

ProC-BPC3 (phs000812.v1.p1):

The Breast and Prostate Cancer Cohort Consortium (BPC3) genome-wide association studies of advanced prostate cancer and estrogen-receptor negative breast cancer was supported by the National Cancer Institute under cooperative agreements U01-CA98233, U01-CA98710, U01-CA98216, and U01-CA98758 and the Intramural Research Program of the National Cancer Institute, Division of Cancer Epidemiology and Genetics.

PanC-PanScan (phs000206.v5.p3):

This project was funded in whole or in part with federal funds from the National Cancer Institute (NCI), US National Institutes of Health (NIH) under contract number HHSN261200800001E. Additional support was received from NIH/NCI K07 CA140790, the American Society of Clinical Oncology Conquer Cancer Foundation, the Howard Hughes Medical Institute, the Lustgarten Foundation, the Robert T. and Judith B. Hale Fund for Pancreatic Cancer Research and Promises for Purple. A full list of acknowledgments for each participating study is provided in the Supplementary Note of the manuscript with PubMed ID: 25086665.

Supplementary References

1. Farha S, *et al.* Hypoxia-inducible factors in human pulmonary arterial hypertension: a link to the intrinsic myeloid abnormalities. *Blood* **117**, 3485-3493, https://doi.org/10.1182/blood-2010-09-306357, (2011).

2. Loirand G, Pacaud P. The role of Rho protein signaling in hypertension. *Nat Rev Cardiol* **7**, 637-647, https://doi.org/10.1038/nrcardio.2010.136, (2010).

3. Wang C. The Relationship between Type 2 Diabetes Mellitus and Related Thyroid Diseases. *J Diabetes Res* **2013**, 390534, https://doi.org/10.1155/2013/390534, (2013).

4. Gowd V, Gurukar A, Chilkunda ND. Glycosaminoglycan remodeling during diabetes and the role of dietary factors in their modulation. *World J Diabetes* **7**, 67-73, https://doi.org/10.4239/wjd.v7.i4.67, (2016).

5. Verges B, Cariou B. mTOR inhibitors and diabetes. *Diabetes Res Clin Pract* **110**, 101-108, https://doi.org/10.1016/j.diabres.2015.09.014, (2015).
