# Supplementary material for: Discovering genetic interactions bridging pathways in genome-wide association studies
Source: Nat Commun. 2019 Sep 19;10:4274. doi: 10.1038/s41467-019-12131-7 (PMC6753138; doi:10.1038/s41467-019-12131-7)
Supplement: Supplementary file 3 — Reporting Summary [file 41467_2019_12131_MOESM3_ESM.pdf]

nature research

Corresponding author(s): Chad Myers, Vipin Kumar, Gang Fang

Last updated by author(s): 2019-06-18

Reporting Summary

Nature Research wishes to improve the reproducibility of the work that we publish. This form provides structure for consistency and transparency in reporting. For further information on Nature Research policies, see Authors & Referees and the Editorial Policy Checklist.

Statistics

For all statistical analyses, confirm that the following items are present in the figure legend, table legend, main text, or Methods section.

n/a	Confirmed	
☐	☒	The exact sample size (n) for each experimental group/condition, given as a discrete number and unit of measurement
☐	☒	A statement on whether measurements were taken from distinct samples or whether the same sample was measured repeatedly
☐	☒	The statistical test(s) used AND whether they are one- or two-sided<br>*Only common tests should be described solely by name; describe more complex techniques in the Methods section.*
☒	☐	A description of all covariates tested
☐	☒	A description of any assumptions or corrections, such as tests of normality and adjustment for multiple comparisons
☒	☐	A full description of the statistical parameters including central tendency (e.g. means) or other basic estimates (e.g. regression coefficient) AND variation (e.g. standard deviation) or associated estimates of uncertainty (e.g. confidence intervals)
☐	☒	For null hypothesis testing, the test statistic (e.g. F, t, r) with confidence intervals, effect sizes, degrees of freedom and P value noted<br>*Give P values as exact values whenever suitable.*
☒	☐	For Bayesian analysis, information on the choice of priors and Markov chain Monte Carlo settings
☒	☐	For hierarchical and complex designs, identification of the appropriate level for tests and full reporting of outcomes
☐	☒	Estimates of effect sizes (e.g. Cohen's d, Pearson's r), indicating how they were calculated

Our web collection on statistics for biologists contains articles on many of the points above.

Software and code

Policy information about availability of computer code

Data collection	No software was used to collect data.
Data analysis	We used BridGE (Bridging Gene Sets with Epistasis) software version 1.01 for discovering the disease-specific pathway-level genetic interactions reported in this paper. The software is available at http://csbio.cs.umn.edu/bridge, which is described in the manuscript. This software is freely available for academic use and non-profit research, and can be licensed for commercial use.

For manuscripts utilizing custom algorithms or software that are central to the research but not yet described in published literature, software must be made available to editors/reviewers. We strongly encourage code deposition in a community repository (e.g. GitHub). See the Nature Research guidelines for submitting code & software for further information.

Data

Policy information about availability of data

All manuscripts must include a data availability statement. This statement should provide the following information, where applicable:

- Accession codes, unique identifiers, or web links for publicly available datasets
- A list of figures that have associated raw data
- A description of any restrictions on data availability

The genome-wide association datasets (PD-NIA, PD-NGRC, SZ-GAIN, BC-CGEMS-EUR, BC-MCS-JPN, BC-MCS-LTN, HT-eMERGE, ProC-CGEMS, ProC-BPC3 and PanC-PanScan) used in this study are available at https://www.ncbi.nlm.nih.gov/gap. Data access is governed by the dbGaP Authorized Access program. The genome-wide association datasets (SZ-GAIN, HT-WTCCC, T2D-WTCCC) used in this study are available at https://www.wtccc.org.uk/. Data access is controlled by the Wellcome Trust Case Control Consortium. The genome-wide association dataset (SZ-CATIE) is available at https://www.nimhgenetics.org/. Data access is controlled by the NIMH Repository and Genomics Resource (NRGR) support team.

Field-specific reporting

Please select the one below that is the best fit for your research. If you are not sure, read the appropriate sections before making your selection.

☒ Life sciences ☐ Behavioural & social sciences ☐ Ecological, evolutionary & environmental sciences

For a reference copy of the document with all sections, see nature.com/documents/nr-reporting-summary-flat.pdf

Life sciences study design

All studies must disclose on these points even when the disclosure is negative.

Sample size	The 12 GWAS datasets used in this study all have at least 500 samples which are sufficient for a typical genotype-phenotype association analysis.
Data exclusions	Samples were excluded if they met any of the following criteria: (1) they had more than a 2% missing genotype rate; (2) were outliers of the reported race group based on multi-dimensional scaling (MDS) analysis when combined with the HapMap reference data; (3) were genetically related to other samples in the same dataset (i.e. IBD > 0.2); or (4) could not be matched to a case (control) in the proposed matched case-control setting based on the genotype clustering analysis described in our methods section.
Replication	For the 7 diseases we analyzed in this study, 4 of them have corresponding independent datasets for testing replication. We reported replication status for each of our discoveries when available.
Randomization	The samples were allocated to case and control groups when we retrieved them from the corresponding data depositories. In our data analysis, we matched the population structure between cases and controls. In the randomization test, we shuffled the samples labels(case/control) while maintaining the matched case-control population structure.
Blinding	The investigators were blinded to group allocation during data analysis.

Reporting for specific materials, systems and methods

We require information from authors about some types of materials, experimental systems and methods used in many studies. Here, indicate whether each material, system or method listed is relevant to your study. If you are not sure if a list item applies to your research, read the appropriate section before selecting a response.

Materials & experimental systems

n/a	Involved in the study	
☒	☐	Antibodies
☒	☐	Eukaryotic cell lines
☒	☐	Palaeontology
☒	☐	Animals and other organisms
☒	☐	Human research participants
☒	☐	Clinical data

Methods

n/a	Involved in the study	
☒	☐	ChIP-seq
☒	☐	Flow cytometry
☒	☐	MRI-based neuroimaging
