# Supplementary material for: Discovering genetic interactions bridging pathways in genome-wide association studies
Source: Nat Commun. 2019 Sep 19;10:4274. doi: 10.1038/s41467-019-12131-7 (PMC6753138; doi:10.1038/s41467-019-12131-7)
Supplement: Supplementary file 4 — Description of Additional Supplementary Files [file 41467_2019_12131_MOESM4_ESM.pdf]

Description of Additional Supplementary Files

Supplementary Data 1: Information about the 13 genome-wide association studies (GWAS) data sets used in this study.

Supplementary Data 2: List of 833 gene sets from KEGG, BioCarta and Reactome.

Supplementary Data 3: BridGE results from PD-NIA cohort based on recessive/dominant combined disease model. BridGE results are reported for the PD-NIA cohort, with the following tabs (in order): summary of discoveries, between-pathway model (BPM) interactions, within-pathway model (WPM) interactions, and hub pathways (pathways exhibiting elevated density of SNP-SNP interactions across the genome) (PATH). Decreased risk (protective) and increased risk (risk) interactions are listed separately. These results were derived using the combined recessive-dominant disease model.

Supplementary Data 4: List of BPMs and WPMs after filtering for redundancy for the PD-NIA cohort. This file contains a list of BPMs obtained from the PD-NIA cohort after controlling for redundancy based on a maximum overlap coefficient of 0.25. These correspond to the set visualized in Fig. 3 of the manuscript.

Supplementary Data 5: Pathway enrichment analysis for single locus effects for PD-NIA. Pathway enrichment analysis on single locus effects was computed for several different disease models and subsets of SNPs. Each of the following tabs appears in this file: (A) combined disease model, LD controlled SNP set, (B) dominant disease model, LD controlled SNP set, (C) recessive disease model, LD controlled SNP set, (D) combined disease model, genome-wide SNP set, (E) dominant disease model, genome-wide SNP set, (F) recessive disease model, genome-wide SNP set.

Supplementary Data 6: Replication statistics and lists of replicated BPMs for BridGE discoveries from PD-NIA. BPMs discovered from the PD-NIA cohort were tested for replication in the independent PD-NGRC cohort. Tab (A) contains a summary of replication statistics and tab (B) contains a list of replicated BPMs.

Supplementary Data 7: Summary of between and within-pathway interactions discovered across six diseases. This file contains a list of BPMs and WPMs (top 10) discovered across six diseases. These correspond to the set visualized in Fig. 5 of the manuscript.

Supplementary Data 8: Summary of interactions discovered across 13 GWAS cohorts. The number of between-pathway model (BPM) interactions, within-pathway model (WPM) interactions, and hub pathways (pathways exhibiting elevated density of SNP-SNP interactions across the genome) (PATH) discovered are reported for each of the 13 GWAS cohorts at a range of FDR cutoffs.

Supplementary Data 9: BridGE results from PD-NGRC cohort based on dominant disease model. BridGE results are reported for the PD-NGRC cohort, with the following tabs (in order): summary of discoveries, between-pathway model (BPM) interactions, within-pathway model (WPM) interactions, and hub pathways (pathways exhibiting elevated density of SNP-SNP interactions across the genome) (PATH). Decreased risk (protective) and increased risk (risk) interactions are listed separately. These results were derived using the dominant disease model.

Supplementary Data 10: BridGE results from SZ-GAIN cohort based on combined disease model. BridGE results are reported for the SZ-GAIN cohort, with the following tabs (in order): summary of discoveries, between-pathway model (BPM) interactions, within-pathway model (WPM) interactions, and hub pathways (pathways exhibiting elevated density of SNP-SNP interactions across the genome) (PATH).

Decreased risk (protective) and increased risk (risk) interactions are listed separately. These results were derived using the combined recessive-dominant disease model.

Supplementary Data 11: BridGE results from SZ-CATIE cohort based on recessive disease model. BridGE results are reported for the SZ-CATIE cohort, with the following tabs (in order): summary of discoveries, between-pathway model (BPM) interactions, within-pathway model (WPM) interactions, and hub pathways (pathways exhibiting elevated density of SNP-SNP interactions across the genome) (PATH). Decreased risk (protective) and increased risk (risk) interactions are listed separately. These results were derived using the recessive disease model.

Supplementary Data 12: BridGE results from BC-MCS-JPN cohort based on dominant disease model. BridGE results are reported for the BC-MCS-JPN cohort, with the following tabs (in order): summary of discoveries, between-pathway model (BPM) interactions, within-pathway model (WPM) interactions, and hub pathways (pathways exhibiting elevated density of SNP-SNP interactions across the genome) (PATH). Decreased risk (protective) and increased risk (risk) interactions are listed separately. These results were derived using the dominant model.

Supplementary Data 13: BridGE results from BC-MCS-LTN cohort based on dominant disease model. BridGE results are reported for the BC-MCS-LTN cohort, with the following tabs (in order): summary of discoveries, between-pathway model (BPM) interactions, within-pathway model (WPM) interactions, and hub pathways (pathways exhibiting elevated density of SNP-SNP interactions across the genome) (PATH). Decreased risk (protective) and increased risk (risk) interactions are listed separately. These results were derived using the dominant model.

Supplementary Data 14: BridGE results from HT-eMERGE cohort based on dominant disease model. BridGE results are reported for the HT-eMERGE cohort, with the following tabs (in order):

summary of discoveries, between-pathway model (BPM) interactions, within-pathway model (WPM) interactions, and hub pathways (pathways exhibiting elevated density of SNP-SNP interactions across the genome) (PATH). Decreased risk (protective) and increased risk (risk) interactions are listed separately. These results were derived using the dominant model.

Supplementary Data 15: BridGE results from HT-WTCCC cohort based on combined disease model. BridGE results are reported for the HT-WTCCC cohort, with the following tabs (in order): summary of discoveries, between-pathway model (BPM) interactions, within-pathway model (WPM) interactions, and hub pathways (pathways exhibiting elevated density of SNP-SNP interactions across the genome) (PATH). Decreased risk (protective) and increased risk (risk) interactions are listed separately. These results were derived using the recessive-dominant combined model.

Supplementary Data 16: BridGE results from ProC-CGEMS cohort based on dominant disease model. BridGE results are reported for the ProC-CGEMS cohort, with the following tabs (in order): summary of discoveries, between-pathway model (BPM) interactions, within-pathway model (WPM) interactions, and hub pathways (pathways exhibiting elevated density of SNP-SNP interactions across the genome) (PATH). Decreased risk (protective) and increased risk (risk) interactions are listed separately. These results were derived using the dominant model.

Supplementary Data 17: BridGE results from ProC-BPC3 cohort based on dominant disease model. BridGE results are reported for the ProC-BPC3 cohort, with the following tabs (in order): summary of discoveries, between-pathway model (BPM) interactions, within-pathway model (WPM) interactions, and hub pathways (pathways exhibiting elevated density of SNP-SNP interactions across the genome) (PATH). Decreased risk (protective) and increased risk (risk) interactions are listed separately. These results were derived using the dominant model.

Supplementary Data 18: BridGE results from T2D-WTCCC cohort based on combined disease model. BridGE results are reported for the T2D-WTCCC cohort, with the following tabs (in order): summary of discoveries, between-pathway model (BPM) interactions, within-pathway model (WPM) interactions, and hub pathways (pathways exhibiting elevated density of SNP-SNP interactions across the genome) (PATH). Decreased risk (protective) and increased risk (risk) interactions are listed separately. These results were derived using the recessive-dominant combined model.

Supplementary Data 19: Replication statistics and lists of replicated BPMs, WPMs or PATHs for BridGE discoveries from prostate cancer, breast cancer and schizophrenia. BPMs, WPMs and PATHs discovered from the each disease cohort were tested for replication in the corresponding independent cohort, for each of the three diseases. Both a summary of replication statistics and a list of replicated BPMs, WPMs or PATHs are reported, with one disease cohort per tab.

Supplementary Data 20: Comparison between BridGE pathways and SNPs reported in the GWAS catalog. Summary of the comparison (A) and list of pathways identified by BridGE with FDR≤0.25 and their association with GWAS SNPs for the six diseases studied: (B) Parkinson's disease, (C) Schizophrenia, (D) Breast cancer, (E) Hypertension, (F) Prostate cancer and (G) Type II diabetes.

Supplementary Data 21: Results of pilot experiments for 13 GWAS cohorts. As described in methods, all 13 cohorts on which BridGE was applied were first explored in pilot runs in which a smaller number of SNP permutations. Based on initial estimates of FDR, the disease model and density combination with strongest statistical significance were run in full. Pilot results from all 13 cohorts are included in this file, one per tab.

Supplementary Data 22: Summary of evaluation of hygeSSI SNP-SNP interactions by a logistic regression-based interaction test.

Supplementary Data 23: BridGE results from PD-NIA cohort based on recessive/dominant combined disease model using 1000 sample permutations. BridGE results are reported for the PD-NIA cohort, with the following tabs (in order): summary of discoveries, between-pathway model (BPM) interactions, within-pathway model (WPM) interactions, and hub pathways (pathways exhibiting elevated density of SNP-SNP interactions across the genome) (PATH). Decreased risk (protective) and increased risk (risk) interactions are listed separately. These results were derived using the combined recessive-dominant disease model.
